# Piezo2 in sensory neurons contributes to inflammation-induced bladder overactivity and is associated with the effects of electroacupuncture

**DOI:** 10.3389/fnins.2026.1886563

**Published:** 2026-08-12

**Authors:** Xu Li, Junwei Hu, Yiyue Dong, Qian Zhou, Yuchen Ying, Zhilin Zhang, Qian Fan, Bingli Chen, Qi-Xiang Song, Wei L. Shen, Kangmin Tang, Yuelai Chen

**Affiliations:** 1 Longhua Hospital, Shanghai University of Traditional Chinese Medicine, Shanghai, China; 2 School of Life Sciences and Technology, ShanghaiTech University, Shanghai, China; 3 School of Rehabilitation Science, Shanghai University of Traditional Chinese Medicine, Shanghai, China; 4 Department of Acupuncture, Yueyang Hospital of Integrated Traditional Chinese and Western Medicine, Shanghai University of Traditional Chinese Medicine, Shanghai, China; 5 Department of Urology, Ren Ji Hospital, Shanghai Jiao Tong University School of Medicine, Shanghai, China

**Keywords:** electroacupuncture, interstitial cystitis/bladder pain syndrome, Piezo2, sensory neuron, void spot assay

## Abstract

**Objectives:**

To assess the effects of varying doses of cyclophosphamide (CYP) on lower urinary tract function in rats, and to further investigate the role of Piezo2 in sensory neurons in a CYP-induced interstitial cystitis/bladder pain syndrome (IC/BPS) model and its potential as a therapeutic target for electroacupuncture (EA).

**Methods:**

Lower urinary tract function was assessed by employing cystometry, external urethral sphincter electromyography, and void spot assay. Bladders were harvested for histological observation. L6-S1 dorsal root ganglia were collected for detection of Piezo2 expression by RNAscope, immunofluorescence staining, and Western blotting. L6-S1 spinal cords were harvested for c-Fos immunofluorescence staining to evaluate activation of spinal neurons.

**Results:**

Five days subsequent to the completion of administration with 50, 60, and 75 mg/kg CYP, bladder function in rats still had not returned to baseline levels. However, the 40 mg/kg dose only caused a reduction in the area under the curve of pressure traces, indicating a change in bladder contractility. Administration with 75 mg/kg CYP did not induce a more severe bladder phenotype than 60 mg/kg CYP. Intrathecal injection of antisense oligonucleotides to knockdown Piezo2 expression in sensory neurons ameliorated bladder overactivity and urethral hyperreflexia in rats with CYP-induced IC/BPS, accompanied by lessened activation of spinal neurons. Aside from that, EA improves lower urinary tract function in rats with cystitis, which may be associated with the downregulation of Piezo2 expression in sensory neurons and the lessened activation of spinal neurons.

**Conclusion:**

Piezo2 in sensory neurons contributes to inflammation-induced bladder overactivity and is associated with the effects of EA. Collectively, targeted inhibition of Piezo2 expression and function may represent a promising novel strategy for managing inflammatory bladder dysfunction, with these data also shedding preliminary light on how EA exerts its therapeutic effects in this condition. On top of that, our study provides novel insights into the characteristics of the CYP-induced IC/BPS model and the establishment of a rat void spot assay protocol.

## 1 Introduction

Interstitial cystitis/bladder pain syndrome (IC/BPS) is a debilitating disorder. It is defined as a chronic inflammatory bladder condition excluding confirmed bacterial urinary tract infection, characterized by urinary frequency accompanied by chronic pelvic pain (Hanno et al., 2015; Khullar et al., 2019; Clemens et al., 2022). The estimated prevalence of IC/BPS is 40.2 per 100,000 individuals in Taiwan, China (Lee et al., 2018), whereas its prevalence ranges from 2.7% to 6.5% in the United States (Berry et al., 2010; Konkle et al., 2012). Though non-life-threatening, persistent urinary urgency causes substantial distress to patients in daily life, social interactions and occupational activities, drastically deteriorating their quality of life and triggering adverse psychological conditions such as anxiety and depression (Chuang et al., 2015; Mahjani et al., 2021; Chen B. et al., 2024). Meanwhile, this disease imposes substantial economic burdens: studies have reported that the average annual healthcare expenditure of patients with diagnosed IC/BPS is 2.0 to 2.4-fold higher than that of age-matched control subjects (Clemens et al., 2008).

Clinical studies have demonstrated that patients with IC/BPS manifest heightened sensations of bladder fullness, urinary urge and pelvic pain at lower bladder volumes relative to healthy controls (van Brummen et al., 2004; Kim et al., 2009; Lai et al., 2014). These observations thereby implicate sensitization of bladder sensory neurons is a pivotal feature of IC/BPS, which excessively initiates the micturition reflex by enhancing afferent mechanosensory activity (Yoshimura et al., 2014; de Groat and Yoshimura, 2015). Normal bladder function, storing urine rhythmically and evacuating urine appropriately, is controlled by a complex mechanical environment. The accurate transmission of mechanical stimuli by sensory neurons to the spinal cord, which triggers the involuntary micturition reflex, and to the brain, which mediates voluntary urination, is critical for normal bladder function (Li et al., 2023). Nonetheless, our understanding of the molecules used by sensory neurons to detect mechanical stimuli, the associated mechanotransduction pathways, and their contributions to the pathophysiology of IC/BPS is only slowly coming into focus. On that account, a pivotal research objective is to understand how mechanosensory activity in sensory neurons drives the development of IC/BPS. The Piezo channel family is a class of non-selective cation channels that open in direct response to mechanical stimulation, comprising two subtypes, Piezo1 and Piezo2 (Coste et al., 2010). These channels act as mechanotransduction molecules in a wide range of cell types to regulate various physiological and pathophysiological processes (Li et al., 2022; Swain and Liddle, 2023; Xiao, 2024). Piezo2 is predominantly expressed in primary sensory neurons and plays a pivotal role in mechanotransduction during physiological processes such as airway stretch, gastrointestinal peristalsis and sexual function (Nonomura et al., 2017; Lam et al., 2023; Servin-Vences et al., 2023). Current research has shown that Piezo2 is involved in the mechanism of neural sensing of bladder volume. Specific knockout of Piezo2 in sensory neurons increases the micturition contraction interval in mice and immensely attenuates the urethral reflex (Marshall et al., 2020). Nevertheless, it remains ambiguous how Piezo2 in sensory neurons is involved in the pathological mechanism of IC/BPS.

As already demonstrated by clinical studies, acupuncture is an effective intervention for lower urinary tract disorders, including stress urinary incontinence, IC/BPS and overactive bladder (OAB) (Yuan et al., 2015; Liu et al., 2017; Bresler et al., 2022; Tang et al., 2023; Noh et al., 2025). Nonetheless, it remains incompletely elucidated with regard to the fundamental principles and mechanisms underlying acupuncture’s therapeutic effects, which severely hinders its broader clinical adoption and dissemination. The Ciliao (BL32) acupoint is one of the most extensively utilized acupoints for electroacupuncture (EA) in clinical practice, and its effect on improving bladder function in animal models of cyclophosphamide (CYP)-induced IC/BPS has been observed (Su et al., 2025; Xiao et al., 2026). Preliminary mechanistic studies suggest that EA at BL32 may alter the firing properties of Barrington’s nucleus and its surrounding bladder activity-related neurons via the GABAergic system, thereby inhibiting bladder contractions (Wang et al., 2012). Results from a recent study have indicated that EA at BL32 alleviates CYP-induced bladder dysfunction and mechanical pain by downregulating the BDNF-TrkB signaling pathway in the dorsal horn of the spinal cord (Su et al., 2025). Furthermore, the beneficial therapeutic effects of EA at BL32 may also be attributed to the restoration of autophagy in spinal inhibitory interneurons (Xiao et al., 2026). These findings underline the paramount role of central neural circuits in the effects of EA. As evidently demonstrated by our recent findings, EA at the BL32 attenuates urothelial mechanotransduction in rats with CYP-induced bladder overactivity, suggesting that the micturition afferent pathway may be a potential mechanism underlying the effects of EA (Junwei et al., 2025). A recent study found that EA reduces Piezo2 expression in sensory neurons of TNBS-induced colitis mouse models. Aside from that, chemogenetic activation of Piezo2-expressing colonic sensory neurons considerably attenuates the therapeutic effects of EA (Chen et al., 2025). Nevertheless, it remains ambiguous with regard to whether EA exerts therapeutic effects on IC/BPS through sensory neurons and whether the underlying molecular mechanisms involve Piezo2.

As our data demonstrated, the expression of Piezo2 channels in sensory neurons was functionally upregulated in rats with CYP-induced IC/PBS. Lower urinary tract function was assessed by utilizing cystometry, electromyography, and a self-developed void spot assay. The results demonstrated that both intrathecal ASO injection and EA intervention downregulated Piezo2 expression in sensory neurons, concomitant with improved lower urinary tract function and attenuated activation of spinal neurons. As these findings suggest, targeted inhibition of Piezo2 expression and function can not only serve as a novel therapeutic approach for IC/BPS, but also provide preliminary insights into the underlying mechanism explaining the efficacy of EA in treating IC/BPS.

## 2 Materials and methods

### 2.1 Animals

The welfare and use of laboratory animals in this study were in line with the institutional guidelines of Shanghai University of Traditional Chinese Medicine. The ethics approval number is PZSHUTCM2502240005. Specific pathogen-free female Sprague-Dawley rats, weighing 200–220 g, were purchased from Beijing Vital River Laboratory Animal Technology Co., Ltd. The laboratory animals were housed in a SPF facility at the Laboratory Animal Center of Shanghai University of Traditional Chinese Medicine. The housing environment was quiet, with a controlled temperature (20°C–24°C) and humidity (40%–50%) in a 12-h reverse light/dark cycle (light time, 7 p.m.–7 a.m.). No more than five rats were kept per cage, with ad libitum access to food and water.

### 2.2 Cyclophosphamide-induced IC/BPS

A fresh solution of CYP (Sigma, PHR1404) was prepared with 0.9% normal saline (NS), and intraperitoneally injected into rats on Day 1, Day 4, and Day 7.

### 2.3 Knockdown Piezo2 with ASO

Attributable to the deficiency of selective inhibitors that directly target the Piezo2, we referred to the study conducted by Ferrari et al. (2015) and adopted the strategy of intrathecal injection of ASO to knock down the level of Piezo2 mRNA in dorsal root ganglion (DRG) neurons. With an aim to maximize the knockdown efficiency, a mixture of ASOs was constructed by utilizing three types of single-stranded ribonucleic acids complementary to the rat Piezo2 mRNA sequence (GenePharma): (1) 5′-CCACCACATAAACACCTGC-3′, (2) 5′-TTCCTCCTCTTCACTATCCG-3′, (3) 5′-CCTCAATG GTTTCCGTAGTTC-3′. Aside from that, 2’-O-methyl and 5′-cholesterol modifications were introduced to reinforce the targeting ability and interference efficiency of the ASOs. For the control experiment, a mismatched ASO mixture was constructed by employing three types of single-stranded ribonucleic acids that do not complement any gene sequence of rats: (1) 5′-ACATCACACGAACTCCAGC-3′, (2) 5′-GTCATCGTCATCACATTGCG-3′; (3) 5′-TCTCAGTGCTCTCC ATAGGTA-3′. Both the knockdown and control experiments involved the intrathecal delivery of ASO or mismatched ASO into the intrathecal space, with the agent formulated at a concentration of 3.5 μg/μl and a total volume of 20 μl per injection. In an effort to achieve optimal knockdown efficiency, intrathecal injections were administered once every other day for a total of six rounds.

### 2.4 Intrathecal injection

After anesthetizing the rat with isoflurane (R510-22-10, RWD), the hair on the lower back was shaved off. Afterwards, the rat was fixed in a prone position on a thermostatic electric blanket, and a 50 ml centrifuge tube was placed on the abdomen to make the L5 spinous process protrude. After disinfection with iodophor, 20 μl of ASO or mismatched ASO was slowly injected into the L4–L5 intervertebral space by employing a microliter syringe (80401, Hamilton) fitted with a 23G needle (0.6 × 25 mm). The needle was inserted at an approximate angle of 70 degrees, and a sudden tail flick in the rats indicated successful penetration into the spinal canal. Subsequent to the above steps, the angle was gradually adjusted to about 45 degrees during injection, and the needle was not withdrawn until 1 min had elapsed subsequent to the full administration of the agent.

### 2.5 EA

The BL32 is located in the second posterior sacral foramen. The non-acupoint points were selected at sites 1 mm lateral to the bilateral BL32. After anesthetizing the rat with isoflurane, a sterile acupuncture needle (0.25 × 25mm) was inserted perpendicularly 15 mm deep into the BL32. For the non-acupoint EA treatment, the needle was inserted perpendicularly only until the tips touched the sacrum, without penetrating into the posterior sacral foramen. The acupoints on both sides were connected to the same pair of output electrodes. The electrical current was set at 1.0 mA, and the stimulation lasted for 20 min, with a pulse width of 200 μs and a frequency of 10 Hz controlled by utilizing a stimulator (FE180, AD Instruments). The intervention was performed once daily for five consecutive days. EA at the non-acupoint sites was performed in the same manner as described above. All interventions were initiated 1 day after CYP administration.

### 2.6 Cystometry and electromyography

After anesthetizing the rat to be tested via intraperitoneal injection of urethane (10%, 1.2 g/kg), a 0.97 × 0.58 mm PE-50 polyethylene catheter (62327, RWD) was inserted into the bladder through a surgical stoma at the dome of the bladder, and then secured with 4-0 nylon sutures. Through a three-way stopcock, the exposed PE-50 catheter was interfaced with two components: a pressure transducer (MLT844, AD Instruments) and a 50 ml syringe mounted on a microinjection pump, with the former being connected to a bridge amplifier (FE221, AD Instruments). Platinum electrodes (771000, A-M system), connected to a biological amplifier (FE234, AD Instruments), were inserted bilaterally into the external urethral sphincter (EUS), with room-temperature liquid paraffin applied topically to maintain urethral moisture. Pre-warmed NS was infused into the bladder at a rate of 6 ml/h, while pressure and electromyography (EMG) signals were recorded at a sampling rate of 1 kHz by utilizing a PowerLab data acquisition system (PL2604, AD Instruments), and data visualization and analysis were performed on LabChart 8. The contraction interval refers to the time interval between two micturition events; the peak bladder pressure represents the maximum intravesical pressure within a single micturition cycle; the bladder pressure baseline denotes the minimum intravesical pressure throughout the filling phase of a micturition cycle; the pre-contraction pressure is defined as the pressure value 5 s prior to the peak bladder pressure; bladder compliance is calculated according to the formula: [(Time corresponding to pre-contraction pressure−Time corresponding to bladder pressure baseline) × Perfusion rate]/(Pre-contraction pressure−Bladder pressure baseline). The area under the curve of pressure traces reflects the amplitude and duration of bladder contractions, which we defined as bladder contractions in this study (Marshall et al., 2020). The parameters quantified in EUS EMG analyses include root mean square, integrated electromyography (iEMG), and bursting duration. The EMG data were first processed with 20 Hz high-pass filtering and 50 Hz notch filtering, followed by iEMG and root mean square analyses performed in LabChart 8 software. Given that surgical manipulation may induce unstable bladder contractions, the above analysis was based on the statistical data of 6 consecutive micturition cycles recorded 1 h subsequent to the start of data collection. Cystometry and EUS EMG was performed either 6 days after CYP administration, or 1 day following the completion of EA/non-acupoint EA interventions.

### 2.7 Void spot assay

The rat to be tested was placed in a custom-made stainless-steel cage (60 × 60 × 26 cm, grid size 1 × 6 cm). The cage was elevated 2 cm above the filter paper (PA1-0.7, SPERIKON) by employing a custom-made support. Filter paper with dimensions of 80 × 80 cm was selected to display the complete imprints of urine spots. Between 8:00 and 12:00 a.m. and under dark conditions, a void spot assay was carried out, with the rat in the cage having free access to food but being deprived of water. For two consecutive days prior to the start of the experiment, the rat was placed in the cage throughout the measurement time period for acclimatization. Upon completion of the assay, the filter paper was air-dried naturally. Subsequently, a fresh ninhydrin (N105630, Aladdin) solution with a concentration of 2 mg/mL was prepared by utilizing anhydrous ethanol, which was then evenly sprayed onto the filter paper until its surface was fully moistened. The filter paper was placed in a drying oven for drying, and finally, images were taken and saved by employing a camera. Apart from that, to quantify the void volume, we collected the rat’s urine and established a standard curve of void spot area versus urine volume. Specifically, a pipette was employed to aspirate volumes of 5, 10, 20, 50, 100, 200, 500, 750, 1,000, 1,500, and 2,000 μl of rat urine, respectively, which were subsequently dropped onto the filter paper from a height of 2 cm. The above procedure was repeated once. After the filter paper was air-dried naturally, the outlines of the urine spots were traced with a pencil and photographed for documentation. The percentage of each urine spot in contrast to the filter paper area (% area) was calculated. This normalization method could eliminate errors stemmed from camera zoom, shooting position, and filter paper deformation after wetting. Afterwards, the standard curve was generated by performing non-linear fitting of the urine output and %area data in GraphPad Prism. The number and total area of urine spots were quantified by employing ImageJ; only those spots larger than 0.25 cm^2^ were included in the statistics. Void volume and average void volume per micturition were calculated by utilizing the established standard curve. Void spot assay was performed either 6 days after CYP administration, or 1 day following the completion of EA/non-acupoint EA interventions.

### 2.8 Retrograde labeling of bladder sensory neurons

The rat was anesthetized with isoflurane. After disinfection with iodophor, the lower abdomen was surgically incised to expose the bladder. A Hamilton microsyringe fitted with a 23G needle was employed to inject 5 μl of the lipophilic membrane dye Dil (20 mg/ml in dimethyl sulfoxide) (D3911, Invitrogen) into five different sites of the bladder wall, respectively. Care was taken to avoid puncturing through to the bladder lumen. The needle was held in place for 20 s and then slowly withdrawn. DiI injection was performed following 7 days of animal adaptive feeding.

### 2.9 Fluorescent in situ hybridization

Upon completion of all functional assessments, the rat still under anesthesia was perfused transcardially with 100 ml of PBS, after which the L6 lumbar DRG tissues were rapidly harvested and fixed in 4% paraformaldehyde for 2 h. After gradient dehydration with 20% and 30% sucrose solutions, the DRG tissues were sectioned into 12 μm-thick slices by employing a cryostat microtome (CM3050s, Leica), and the slices were directly mounted onto adhesive slides. Subsequently, the procedure was performed in accordance with the protocol for fixed frozen sections provided in the monochromatic fluorescent RNA in situ hybridization kit (PIF1000, Pinpoease). Probes for Piezo2 (3073801-C2, Pinpoease) and the fluorescent substrate PIN 488 (PIF0002, Pinpoease) were applied to detect transcript. After mounting the sections with DAPI Fluoromount-G mounting medium (0100-20, SouthernBiotech), the slides were observed under an upright fluorescence microscope (Axio Imager 2, Zeiss), and all sections were scanned and saved by employing virtual microscopy slide scanning system (VS120-S6-W, Olympus). The average fluorescence intensity of the green channel in bladder sensory neurons labeled with Dil red fluorescence was measured by utilizing ImageJ. In an effort to avoid duplicate counting, only neurons with visible intact nuclei were analyzed.

### 2.10 Immunofluorescence

The immediate early gene c-Fos, a transcription factor, was used as a marker of neuronal activity to characterize the activation status of micturition-related spinal cord neurons stimulated by afferent signals. As reported by Birder and de Groat (1992), maximal expression of c-Fos in the spinal cord 2 h after the intravesical infusion of saline. Therefore, the spinal cord and DRG tissues were harvested immediately following 2 h of continuous intravesical saline perfusion and fixed in 4% paraformaldehyde at 4°C for 18 and 2 h, respectively. Tissues were dehydrated in sucrose (20% and 30%, 2 days) at 4°C, and then sectioned at 35 and 12 μm thickness on a cryostat microtome, respectively. Spinal cord sections were collected in PBS-containing plates, whereas DRG sections were directly attached to adhesive slides. Block with immunostaining blocking buffer for 1.5 h. For DRG sections, incubate them with rabbit anti-Piezo2 primary antibody (1:200, NBP1-78624, Novus Biologicals; primary antibody dilution buffer, P0262, Beyotime) overnight at 4°C, followed by incubation with Alexa Fluor™ 488-conjugated goat anti-rabbit IgG secondary antibody (1:500, A11008, Invitrogen; secondary antibody dilution buffer, P0108, Beyotime) for 2 h at room temperature (26°C). Spinal cord sections were incubated overnight at 4°C with guinea pig anti-Fos primary antibody (1:200, 226308, Synaptic Systems), after which they were incubated for 2 h at room temperature with Alexa Fluor™ 647-conjugated goat anti-guinea pig IgG secondary antibody (1:500, 33413ES60, Yeasen). Then, the sections were mounted on coverslips with DAPI Fluoromount-G mounting medium. The sections were washed three times (10 min per wash) in PBS subsequent to fixation, sucrose dehydration and the two rounds of incubation. For spinal cord sections, only the number of c-Fos-positive neurons in the dorsal horn region was counted. For DRG sections, ImageJ was employed to analyze the mean green fluorescence intensity of bladder sensory neurons labeled with Dil red fluorescence.

### 2.11 Western blotting

The L6-S1 DRG tissues were lysed in RIPA buffer for protein extraction. After being fractionated by SDS-PAGE, the protein samples were electrotransferred onto PVDF membranes. After blocking with western blocking buffer (P0252, Beyotime) for 30 min at room temperature, the PVDF membranes were incubated with rabbit anti-Piezo2 antibody (1:1000, NBP1-78624, Novus Biologicals; primary antibody dilution buffer, P0023A, Beyotime) and mouse anti-β-actin antibody (1:5000, MA1-140, Invitrogen) overnight at 4°C and washed for three times in 0.1% TBST, then sequentially incubated with goat anti-rabbit IgG HRP-conjugated secondary antibody (1:5000, 31460, Invitrogen; secondary antibody dilution buffer, P0258, Beyotime) and goat anti-mouse IgG HRP-conjugated secondary antibody (1:5000, 31430, Invitrogen) at room temperature for 1 h. Antigen-antibody complexes were detected by utilizing an ECL chemiluminescence detection kit (HY-K2005, MedChemExpress), and images were captured and archived with a multi-functional gel image analysis system (MINI Space 1000, Tanon). Protein expressions were quantified by employing ImageJ.

### 2.12 Statistical analysis

All the raw data were performed by employing Excel 2021, and were subsequently imported into GraphPad Prism (v.10.2.3) for statistical analysis and visualization. Heatmaps were generated in Matlab (v.2018b). All data were shown as mean ± SEM. Two-tailed paired-samples *t*-test was used for comparisons of body weight before and after CYP treatment. One-way ANOVA followed by post hoc Tukey’s test was employed for all remaining data analyses. A value of *P* < 0.05 was considered statistically significant. All *F*-values and *P*-values are reported in the Supplementary Table.

## 3 Results

### 3.1 Effects of varying doses of cyclophosphamide on lower urinary tract function in rats

**FIGURE 1:**
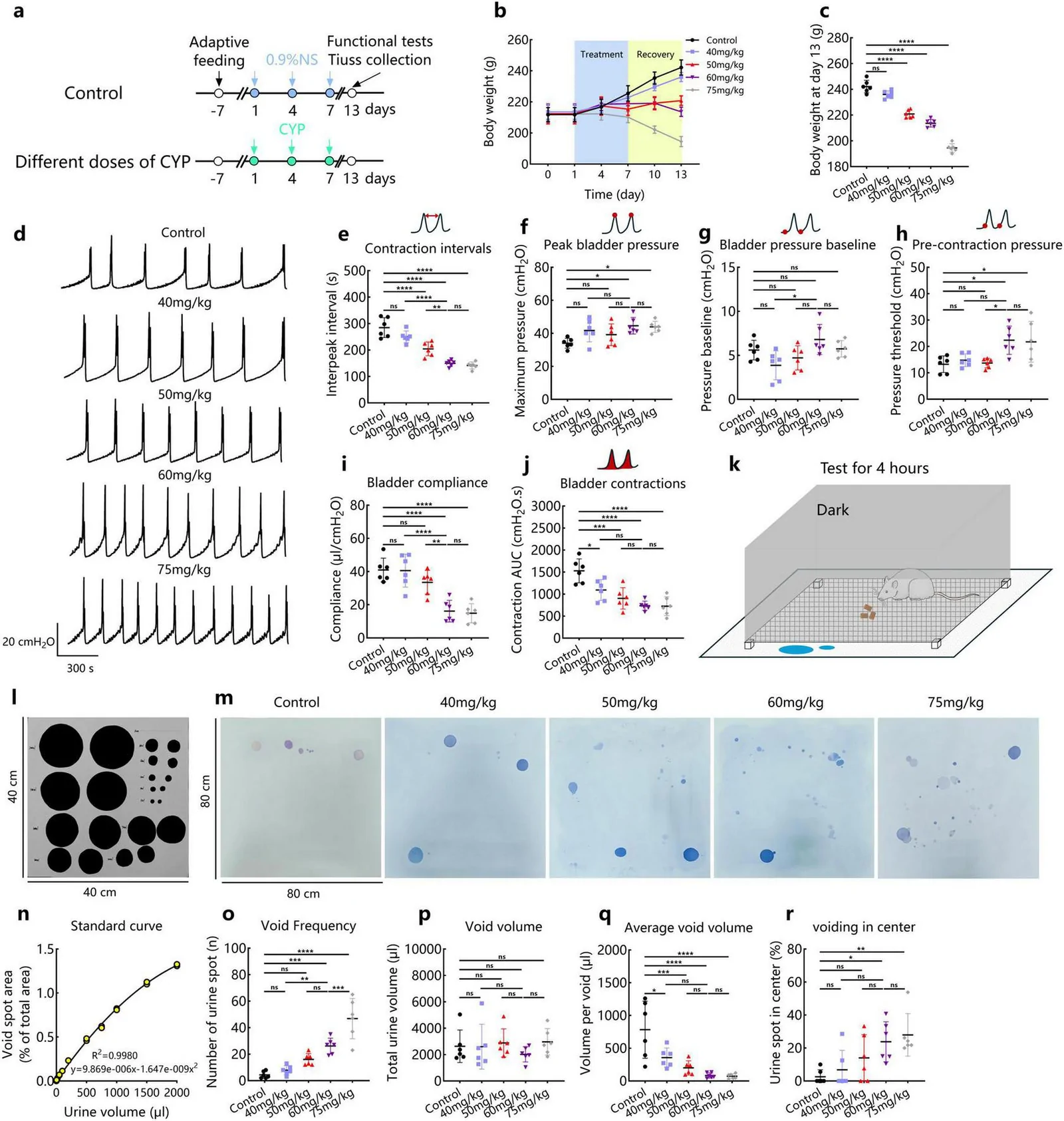
Effects of varying doses of CYP on body weight, and lower urinary tract function in rats. **(a)** Time points for CYP injection and functional measurements. **(b,c)** Trends in body weight changes of rats after intraperitoneal injection of different doses of CYP **(b)**, and comparison of body weight between CYP groups and the control group after injection **(c)**, *n* = 6. **(d)** Example pressure traces of cystometry in rats after administration of different doses of CYP. **(e–j)** Differences in the effects of varying doses of CYP on contraction intervals **(e)**, peak bladder pressure **(f)**, bladder pressure baseline **(g)**, pre-contraction pressure **(h)**, bladder compliance **(i)**, and bladder contractions **(j)** in rats, *n* = 6. **(k)** Void spot assay was performed to evaluate urination patterns in rats. **(l)** Representative images of standard curves generated from collected rat urine samples. **(m)** Representative images of urination patterns in rats after administration of different doses of CYP. **(n)** Standard curves of urine volume and %area obtained by non-linear fitting. **(o–r)** Differences in the effects of varying doses of CYP on void frequency **(o)**, void volume **(p)**, average void volume **(q)**, and void frequency in the middle 50% of the cage **(r)** in rats, *n* = 6. All data are expressed as mean ± SEM. Statistical significance in the graphs is indicated as *P < 0.05, **P < 0.01, ***P < 0.001, ****P < 0.0001.

Cyclophosphamide is the most extensively utilized drug to induce IC/BPS in laboratory animals, and its metabolite acrolein can cause damage to the bladder urothelium, bringing about bladder hyperactivity and pelvic hypersensitivity (Lee et al., 2014). Aside from that, heightened excitability of sensory neurons that innervate the bladder in rodents can be induced by chronic CYP treatment (Boudes et al., 2011; Auge et al., 2020). It is noteworthy that the majority of current studies adopt a chronic dose regimen of (40–75 mg/kg) to establish an IC/BPS model (Auge et al., 2020; Chen B. et al., 2024; Luo et al., 2025; Piao et al., 2025), whereas few studies have reported changes in lower urinary tract function throughout the recovery period after chronic administration. For this reason, we investigated the changes in lower urinary tract function of rats following chronic administration of CYP at different doses (Figure 1a). At 5 days post-termination of administration, rats receiving 50, 60, or 75 mg/kg of CYP exhibited considerably lower body weight in contrast to those injected with 0.9% NS; notably, the 75 mg/kg CYP dose induced an 8% loss of body weight in rats (Figures 1b, c and Supplementary Figure 1a). As cystometry findings demonstrated, rats still exhibited subbaseline bladder function 5 days following the end of injection with 50, 60, and 75 mg/kg of CYP, characterized by lessened contraction intervals and impaired bladder contractility (Figures 1d, e, j). These observations correlated well with pathological results, as evidenced by attenuated bladder urothelial thickness, lamina propria edema and exacerbated fibrosis in the rats (Supplementary Figures 1b–e). On top of that, the 60 and 75 mg/kg doses also gave rise to elevated peak bladder pressure and pre-contraction pressure, as well as lowered bladder compliance (Figures 1f, h, i). With respect to baseline pressure, no remarkable distinctions were observed in rats across the different CYP dose groups in comparison with the 0.9% NS group (Figure 1g). In addition, non-voiding contractions with pressure rises exceeding 5 cmH_2_O were only observed in a small subset of rats. This phenomenon may be attributed to the gradual recovery of CYP-induced bladder injury within 5 days, or alternatively to motion artifacts caused by intravesical PE catheter implantation. As illustrated by void spot assay, 5 days after CYP administration at 60 and 75 mg/kg, rats continued to present with abnormal urination patterns, namely elevated voiding frequency and lessened average void volume (Figures 1k–q). Moreover, more frequent voiding events were observed in the central region of the filter paper among these rats (Figures 1m, r), which confirms the occurrence of incontinence. Nevertheless, administration of CYP at 75 mg/kg did not result in more severe bladder pathological changes in contrast to the 60 mg/kg dose (Supplementary Figures 1b, c), nor were notable discrepancies in bladder phenotypes identified (Figures 1e–j), with only elevated voiding frequency in awake rats being monitored (Figure 1o). Nevertheless, considering that high doses caused body weight loss in rats, a CYP dose of 60 mg/kg was thus adopted for establishing the IC/BPS model in subsequent studies. It is worth noting that the 40 mg/kg dose only caused a reduction in the area under the curve of pressure traces (Figure 1j), indicating a change in bladder contractility.

### 3.2 The expression of Piezo2 in bladder sensory neurons was functionally upregulated in CYP-induced IC/BPS

**FIGURE 2:**
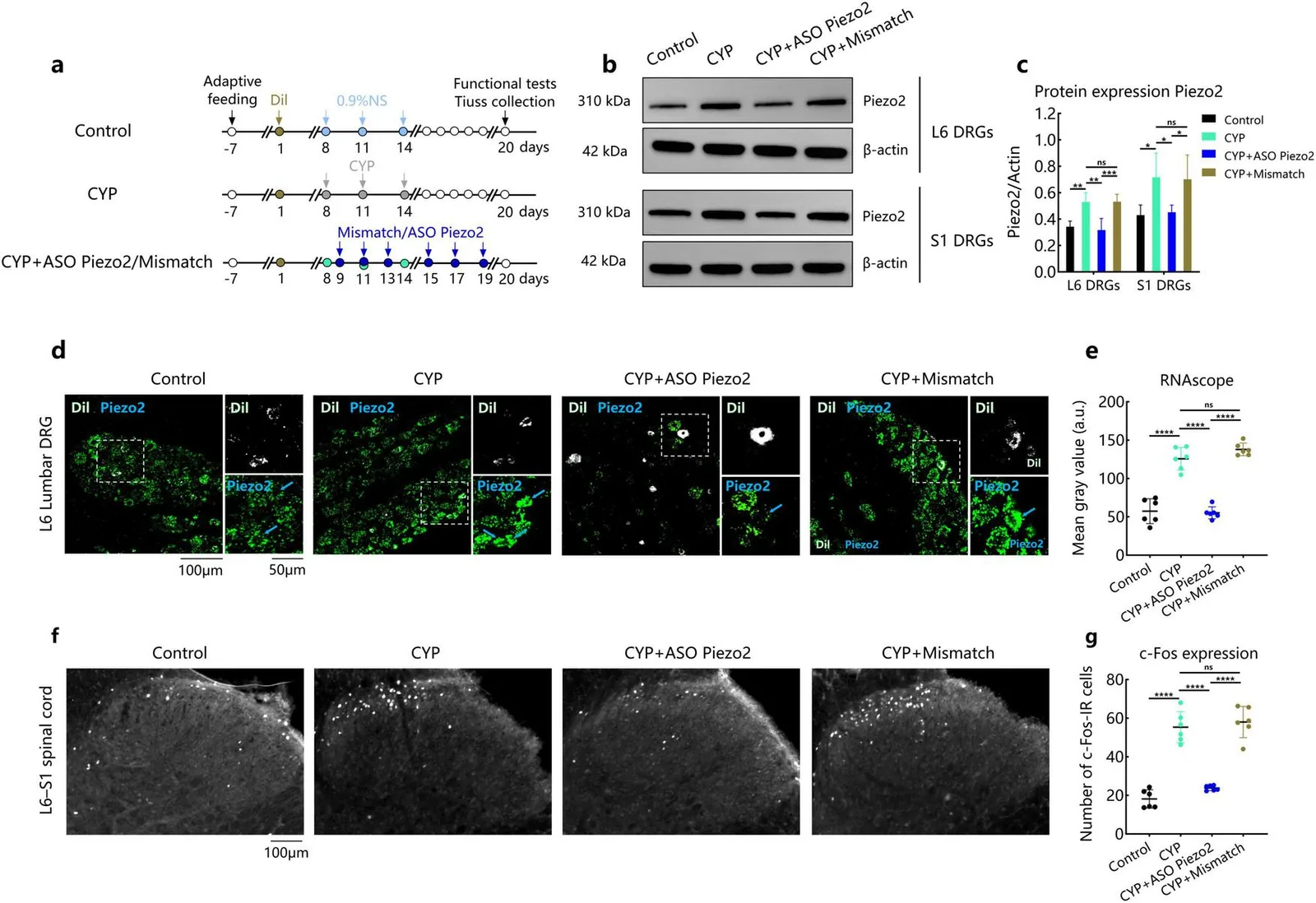
Intrathecal injection of ASO knockdown Piezo2 expression in sensory neurons of rats with IC/BPS. **(a)** Time points for intravesical Dil injection, intraperitoneal CYP injection, intrathecal ASO/Mismatch injection, and functional measurements. **(b)** Assessment of Piezo2 protein expression levels in L6–S1 DRG tissues of rats in each group by western blotting. **(c)** Intrathecal ASO injection significantly reduced Piezo2 protein expression in L6–S1 DRG tissues of rats with IC/BPS. **(d,e)** Evaluation of Piezo2 transcript levels in bladder sensory neurons of rats in each group by RNAscope **(d)**, with quantitative analysis **(e)**. **(f,g)** Representative images of c-Fos immunofluorescence staining in the dorsal horn of the L6-S1 spinal cord of rats in each group **(f)**, with statistical analysis **(g)**. For western blotting, three biological replicates were performed. For each replicate, DRG tissues dissected from five individual rats were pooled per experimental group. All data are expressed as mean ± SEM. Statistical significance in the graphs is indicated as *P < 0.05, **P < 0.01, ***P < 0.001, ****P < 0.0001.

Specific knockout of Piezo2 in sensory neurons gave rise to prolonged contraction intervals and attenuated urethral reflexes in mice, suggesting impaired bladder volume regulation function (Marshall et al., 2020). It can be reasonably hypothesized that Piezo2 overexpression triggers an enhanced sensitivity of sensory neurons to filling in the setting of bladder hyperactivity. On that account, we assessed the association between CYP-induced IC/BPS and alterations in Piezo2 expression levels in sensory neurons (Figure 2a). After injecting the Dil dye into the bladder wall, we collected the DRG tissues, and then used RNA fluorescence in situ hybridization to assess changes in the Piezo2 transcript. Strikingly elevated Piezo2 transcript levels were detected in Dil-labeled bladder sensory neurons of rats receiving CYP administration (Figures 2d, e). Results from western blotting and immunofluorescence staining demonstrated tremendously elevated Piezo2 protein levels in the DRG tissues of CYP-administered rats in contrast to the 0.9% NS-treated controls (Figures 2b, c and Supplementary Figures 1f, g).

**FIGURE 3:**
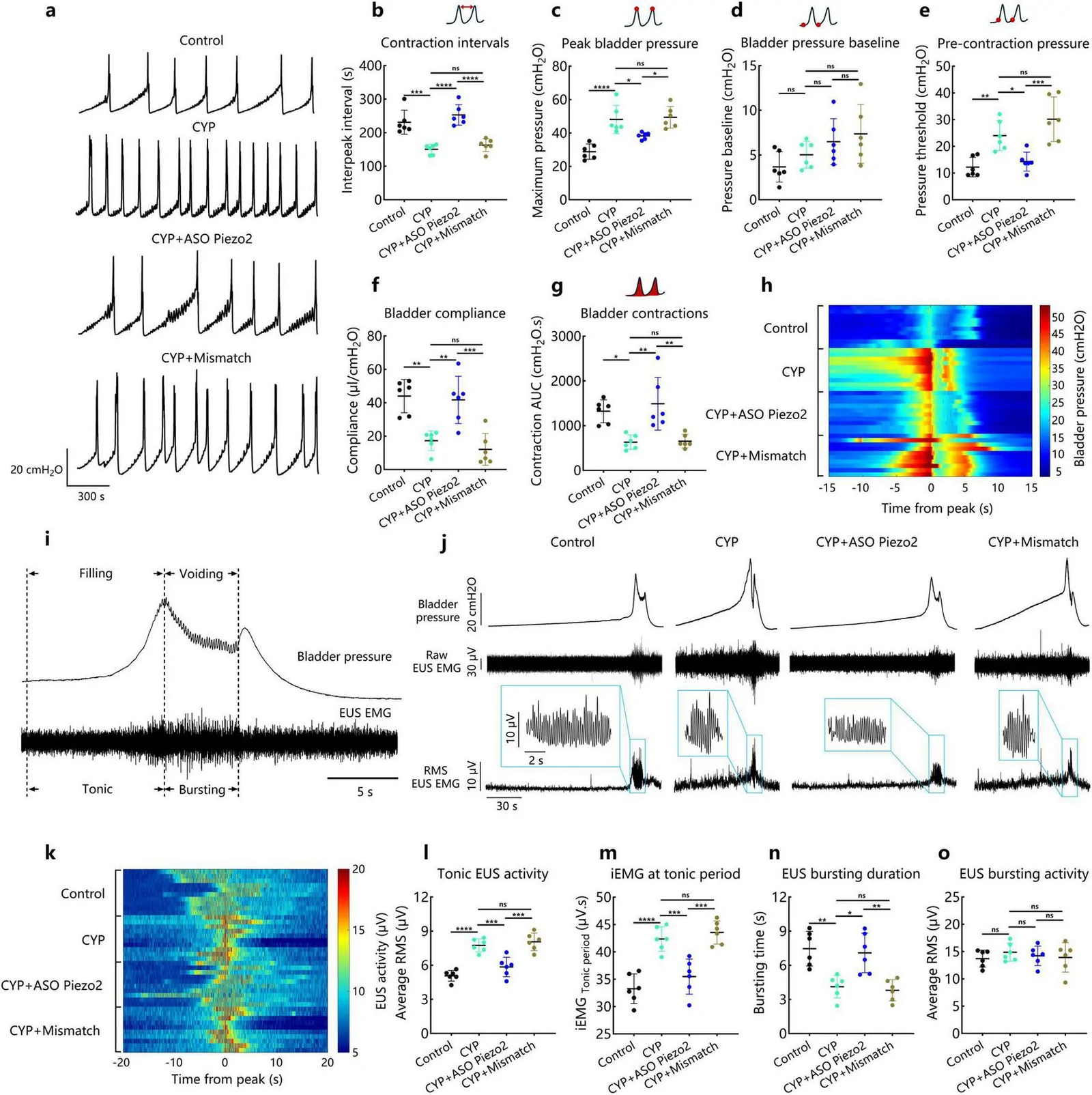
Knockdown of Piezo2 expression in bladder sensory neurons improves lower urinary tract function in rats with IC/BPS. **(a–g)** Example pressure traces of cystometry in rats from each group **(a)**, and comparative analyses of contraction intervals **(b)**, peak bladder pressure **(c)**, bladder pressure baseline **(d)**, pre-contraction pressure **(e)**, bladder compliance **(f)**, and bladder contractions **(g)**, *n* = 6. **(h)** Heatmap showing bladder pressure changes during cystometry in rats from each group. **(i)** Tonic EUS activity converts to marked EUS bursting activity at the onset of voiding contractions. **(j)** Representative images of EUS EMG activity in rats from each group. **(k)** Heatmap showing EUS EMG activity in rats from each group. **(l–o)** Statistical analyses of tonic EUS activity **(l)**, iEMG at tonic period **(m)**, EUS bursting duration **(n)**, and EUS bursting activity **(o)** of EUS EMG in rats from each group, *n* = 6. All data are expressed as mean ± SEM. Statistical significance in the graphs is indicated as *P < 0.05, **P < 0.01, ***P < 0.001, ****P < 0.0001.

Afterwards, we investigated whether the upregulation of Piezo2 expression in bladder sensory neurons was functional. Attributable to the deficiency of selective inhibitors that directly target the Piezo2 channel, we adopted the strategy of intrathecal injection of ASO to knock down the Piezo2 mRNA level in DRG neurons. As confirmed by a sequence of studies, this strategy is capable of suppressing both protein expression and mRNA levels in peripheral neurons. Successful knockdown of Piezo2 protein expression and mRNA levels in bladder sensory neurons was achieved in rats with IC/BPS via intrathecal ASO administration, whereas no such effect was observed in rats receiving mismatched ASO treatment (Figures 2b–e and Supplementary Figures 1f, g). Knock-down of Piezo2 expression in bladder sensory neurons prolonged contraction intervals, lowered peak bladder pressure, heightened bladder compliance, and improved bladder contractility in CYP-administered rats (Figures 3a–h). Void spot assay revealed discrepancies in both voiding frequency and voiding counts in the central region of the filter paper (Supplementary Figures 1h–l). Of note, diminished Piezo2 levels in bladder sensory neurons elicited robust suppression of c-Fos expression in the L6–S1 spinal dorsal horn (Figures 2f, g), which reflects a lessened population of micturition-relevant spinal neurons activated by bladder afferent signaling. As a consequence, the expression of Piezo2 in sensory neurons is elevated in rats with CYP-induced IC/BPS, with knockdown of Piezo2 expression bringing about improvements in bladder function and aberrant urination behavior.

### 3.3 Down-regulation of Piezo2 expression in sensory neurons could reduce urethral activity in rats with IC/BPS

The micturition reflex triggered by afferent activation induces detrusor contraction and urethral sphincter relaxation, which is essential for efficient micturition. On that account, we further assessed whether Piezo2-mediated afferent hyperexcitability in sensory neurons contributes to the enhanced urethral activity in rats with IC/BPS. Consistent with previous findings (Chang et al., 2019), tonic EUS activity throughout bladder filling was converted to prominent EUS bursting activity at the onset of micturition contractions (Figures 3i, j). Tonic EUS activity was tremendously elevated in rats with CYP-induced IC/BPS, with the iEMG value markedly elevated within 5 s prior to micturition contractions (Figures 3j–n), suggesting the activation of more motor units. Apart from that, the duration of bursting activity was immensely lowered (Figures 3j, k, n), which reveals lessened relaxation of the EUS and impaired micturition efficiency. Nonetheless, no striking disparity in EUS bursting activity was observed between CYP-induced IC/BPS model rats and control rats (Figure 3o; Wu et al., 2018), this may represent one of the pathological features throughout the chronic recovery phase. Intrathecal ASO injection in rats with IC/BPS gave rise to attenuated tonic EUS activity, lowered iEMG values, and prolonged bursting activity duration, in contrast to the deficiency of such effects with mismatched ASO administration (Figures 3j–n). These suggest that knocking down Piezo2 expression in sensory neurons can mitigate the elevated urethral activity stemmed from afferent hyperactivity.

### 3.4 EA at BL32 can improve lower urinary tract function in rats with IC/BPS

**FIGURE 4:**
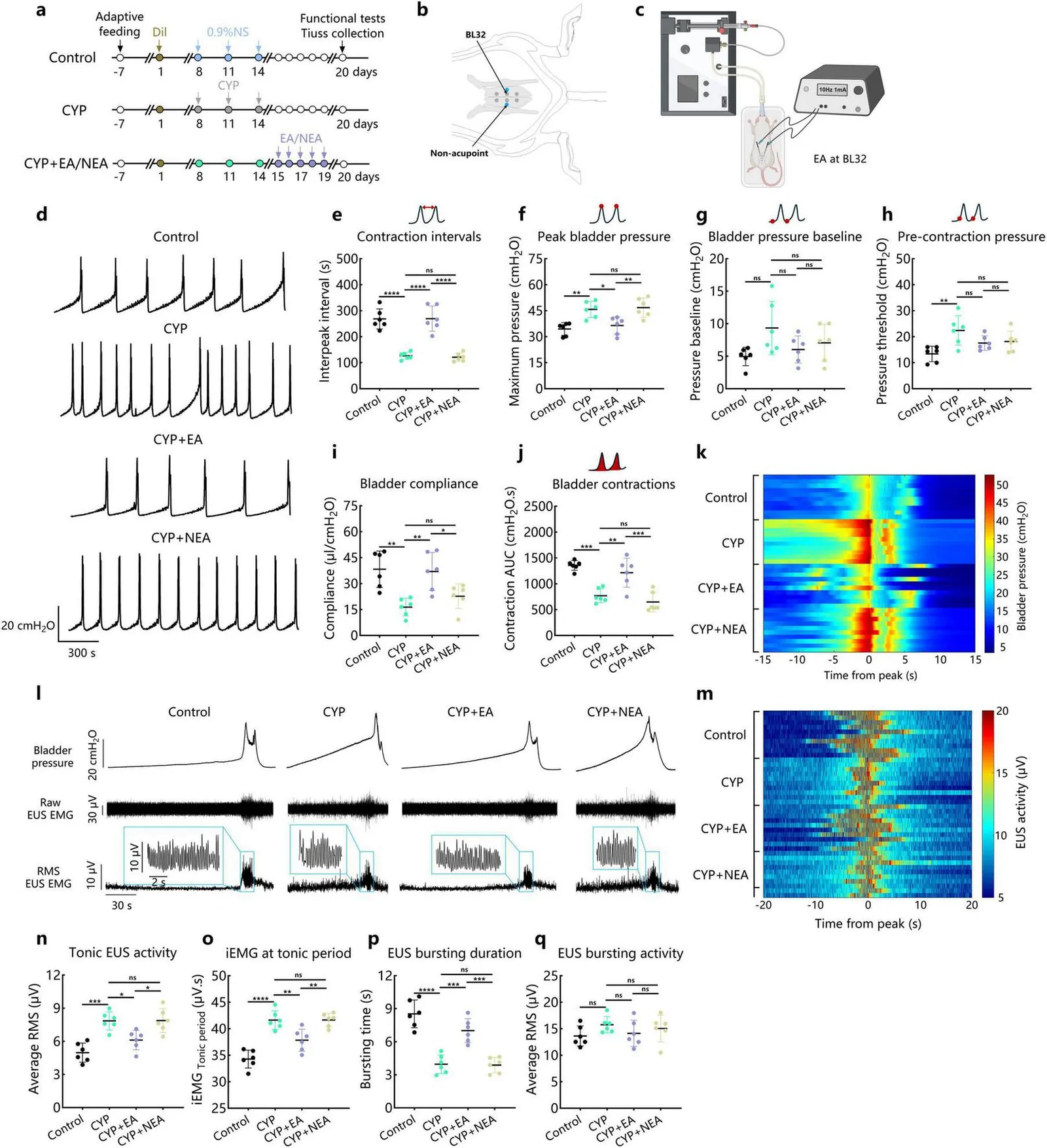
EA at BL32 improves lower urinary tract function in rats with IC/BPS. **(a)** Experimental protocol for each group. **(b,c)** Localization diagram of BL32 acupoint and non-acupoint **(b)**, as well as a schematic diagram of EA procedure **(c)**. **(d–j)** Example pressure traces of cystometry in rats from each group **(d)**, and comparative analyses of contraction intervals **(e)**, peak bladder pressure **(f)**, bladder pressure baseline **(g)**, pre-contraction pressure **(h)**, bladder compliance **(i)**, and bladder contractions **(j)**, *n* = 6. **(k)** Heatmap showing bladder pressure changes during cystometry in rats from each group. **(l)** Representative images of EUS EMG activity in rats from each group. **(m)** Heatmap showing EUS EMG activity in rats from each group. **(n–q)** Statistical analyses of tonic EUS activity **(n)**, iEMG at tonic period **(o)**, EUS bursting duration **(p)**, and EUS bursting activity **(q)** of EUS EMG in rats from each group, *n* = 6. All data are expressed as mean ± SEM. Statistical significance in the graphs is indicated as *P < 0.05, **P < 0.01, ***P < 0.001, ****P < 0.0001.

Clinical studies have demonstrated that electroacupuncture is an effective therapeutic approach for lower urinary tract disorders, including stress urinary incontinence and IC/BPS. The second posterior sacral foramen is the anatomical location of BL32 (Figure 4b), which is counted among the most frequently applied acupoints. In such case, we assessed the effect of EA at bilateral BL32 on lower urinary tract function in rats with IC/BPS, with a non-acupoint control group included for comparison (Figures 4a–c). As demonstrated by cystometry, EA could modulate multiple bladder function parameters in rats with IC/BPS, including the prolongation of contraction intervals, reduction of peak bladder pressure, elevation of bladder compliance and enhancement of bladder contractility (Figures 4d–k). On top of that, this intervention also brought about a marked reduction in void frequency of conscious rats, whereas void volume, average void volume and central-zone void frequency remained unaltered (Supplementary Figures 2a–e). No improvement in lower urinary tract function was detected in rats with IC/BPS receiving EA at non-acupoint points (Figures 4d–k and Supplementary Figures 2a–e). A previous study has reported that Piezo2 knockdown in mouse colonic tissue eliminated EA-induced relief of somatic hypersensitivity accompanying 2,4,6-trinitrobenzene sulfonic acid-triggered colonic inflammation (Guo et al., 2022). Accordingly, we further evaluated whether such an effect could be reproduced in the current experimental model. Our results demonstrated that combined intervention of Piezo2 knockdown in bladder sensory neurons and EA also ameliorated lower urinary tract function in rats with IC/BPS (Supplementary Figures 3a–l). In addition, this combined intervention strategy exerted no significantly superior therapeutic effects compared with monotherapy (Supplementary Figures 3a–l).

We also assessed the effect of EA on urethral activity in rats with IC/BPS. After EA intervention, model rats exhibited attenuated tonic EUS activity, lowered iEMG values, and prolonged bursting activity duration (Figures 4l–q). Nonetheless, this effect was not observed with EA at non-acupoint sites (Figures 4l–q). As demonstrated by these findings, EA at bilateral BL32 can ameliorate lower urinary tract function in rats with IC/BPS.

### 3.5 The therapeutic effects of EA may correlate with the downregulation of Piezo2 in bladder sensory neurons

**FIGURE 5:**
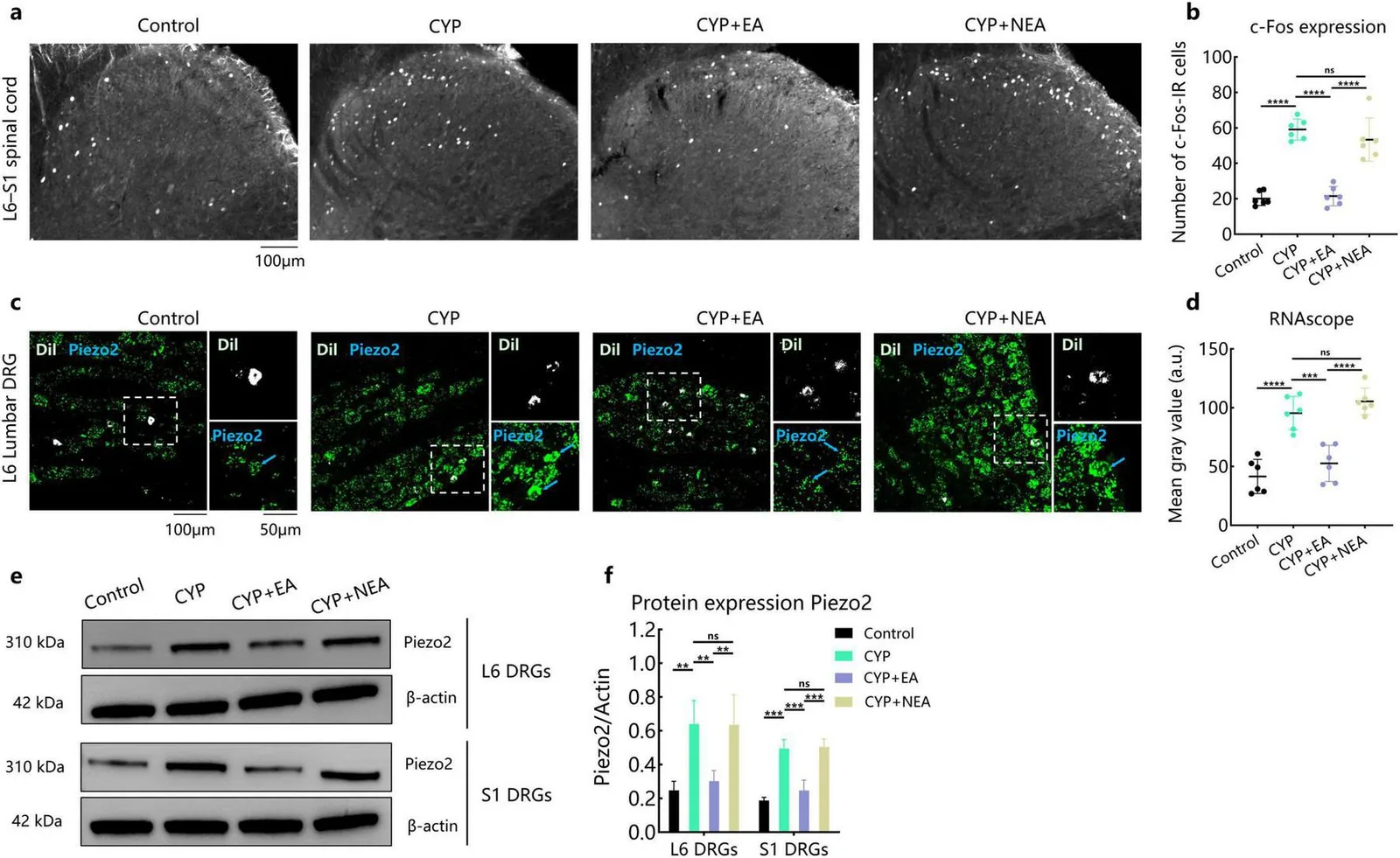
The therapeutic effects of EA may correlate with the downregulation of Piezo2 in bladder sensory neurons. **(a,b)** Representative images of c-Fos immunofluorescence staining in the dorsal horn of the L6-S1 spinal cord of rats in each group **(a)**, and quantitative analysis **(b)**. **(c,d)** Representative images of Piezo2 transcript levels in bladder sensory neurons of rats in each group detected by RNAscope **(c)**, and quantitative analysis **(d)**. **(e,f)** Representative images of Piezo2 protein western blotting in L6–S1 DRG tissues of rats in each group **(e)**, and quantitative analysis **(f**). All data are expressed as mean ± SEM. Statistical significance in the graphs is indicated as *P < 0.05, **P < 0.01, ***P < 0.001, ****P < 0.0001.

Since the posterior divisions of the S1 sacral nerves, which incorporate bladder afferent nerves, pass through the deep tissue at the BL32 in rats, we speculate that the EA-induced effects may involve bladder neural circuits. In contrast to the effect of EA at non-acupoint sites, EA at BL32 markedly downregulated c-Fos expression in the dorsal horn of the spinal cord of rats with IC/BPS (Figures 5a, b), suggesting a lessened population of micturition-associated spinal neurons activated by bladder afferent inputs. On top of that, RNAscope demonstrated that EA lessened the Piezo2 transcript level in bladder sensory neurons (Figures 5c, d); meanwhile, immunofluorescence and western blotting assays demonstrated that Piezo2 protein expression was considerably downregulated in the L6-S1 DRG tissues (Figures 5e, f and Supplementary Figures 2f, g). As suggested by these findings, the beneficial effect of EA at BL32 on lower urinary tract function in rats with IC/BPS is associated with the downregulation of Piezo2 expression in sensory neurons.

## 4 Discussion

The Piezo2 channel is predominantly expressed in primary sensory neurons, and its role in the pathophysiology of the lower urinary tract has been highlighted in recent studies (Marshall et al., 2020). Our results showed that the expression of the Piezo2 channel in bladder sensory neurons was considerably upregulated in a CYP-induced rat model of IC/BPS. Targeted knockdown of Piezo2 expression in sensory neurons alleviated bladder dysfunction and attenuated urethral activity in rats with IC/BPS. Aside from that, the ameliorative effect of EA at BL32 on lower urinary tract function may be associated with the Piezo2 channel. We also provided additional insights into understanding the characteristics of the CYP-induced IC/BPS model and establishing a protocol of void spot assay for rat.

Several animal models have been developed for the study of IC/BPS, including autoimmune cystitis models, bladder inflammation models, Pelvic organ cross sensitization models, and psychological stress (Saito et al., 2025). Inflammation-induced models trigger acute/chronic cystitis via urothelial injury resulted from the injection or intravesical instillation of chemical agents, and a prominent characteristic of these models is the enhanced sensory activity of bladder afferent nerves (Parsons and Drake, 2011; Auge et al., 2020). CYP serves as the most frequently used chemical agent, and the standard chronic dosing regimen for establishing inflammatory IC/BPS models consists of three injections with a single dose of 40–75 mg/kg (Auge et al., 2020; Chen B. et al., 2024; Luo et al., 2025; Piao et al., 2025). In the pilot experiments, we found that the administration of 75 mg/kg, the most commonly reported dose in the literature, gave rise to severe weight loss in rats and even led to mortality throughout the subsequent intervention period. On that account, the present study compared the changes in lower urinary tract function of rats throughout the recovery phase following chronic administration of CYP at different doses. As illustrated by our research findings, the rats exhibited thinning of the bladder urothelium, edema of the lamina propria, and more severe fibrosis 5 days after chronic administration of CYP at doses of 50, 60, and 75. Consistent with these pathological features, functional experiments demonstrated that these rats still presented with bladder hyperactivity, characterized by shortened contraction intervals and enhanced bladder contractility, whereas the bladder function of rats receiving the 40 mg/kg dose basically recovered to the baseline level. This also enabled us to exclude the potential impact of spontaneous recovery on the effects of ASO treatment and EA in subsequent experiments. Notwithstanding the fundamental fact that no rat mortality was observed throughout the experiment, the 75 mg/kg dose was found to induce severe weight loss in rats. Given that the cytotoxic effect of CYP is systemic, such as potent immunosuppressive effects and digestive system toxicity, it may give rise to more extensive organ damage (Lee et al., 2014). Aside from that, we also observed no striking disparity in the degree of bladder pathological changes and bladder functional impairment induced by the 60 mg/kg and 75 mg/kg doses. For this reason, these findings indicate that the 60 mg/kg dose is more suitable for establishing a rat model of IC/BPS.

Similar to the voiding diaries used in clinical practice, the void spot assay is a prevalent approach for evaluating urination behavior patterns in mice (Chen et al., 2017; Hill et al., 2018; Dalghi et al., 2023). Mice are placed in cages lined with filter paper at the bottom, allowed to move freely for a specified period of time, and any micturition event is recorded in the form of urine spots on the paper. The filter paper is subsequently imaged to visualize the urine spot imprints. The biological information provided by the void spot assay includes the number of urine spots, void volume, average void volume, and spatial distribution of urine spots (Dalghi et al., 2023). Nevertheless, there are few reports on the protocols of the void spot assay specifically for rats. In this case, we offer profound insights for establishing a standardized void spot assay protocol in rats. With an aim to prevent rat from gnawing the filter paper, the cages were elevated by 2 cm using custom-made support. Given the physiological tendency of rats to void at the cage edges, filter paper larger than the cage bottom area was employed to capture complete urine spot imprints. Nonetheless, the large size of the filter paper posed challenges for ultraviolet imaging with a conventional gel imager. On that account, ninhydrin solution spraying was adopted for visualization. A recent study reported a substantial distinction in the count of 0–0.1 cm^2^ urine spots between ninhydrin and ultraviolet imaging, and we similarly observed numerous tiny urine spots on filter papers from normal rats (Ruetten et al., 2025). This complicated the accurate counting of genuine small urine spots. Since rats had no direct contact with the filter paper, these imprints could not be footprints, and we hypothesize they may result from dust or inherent characteristics of the filter paper itself. Our optimized protocol revealed dose-dependent effects of CYP on rat urination patterns. Consistent with observations in mice, high-dose CYP tremendously elevated voiding frequency in rats, particularly in the central region of the filter paper (Clayton et al., 2022; de Oliveira et al., 2022), suggesting urinary incontinence. It remains ambiguous about whether this phenotype is attributed to prolonged residence of CYP-administered rats in the central cage area. Under such circumstance, long-term video monitoring in future studies may be advantageous for us to rule out this possibility.

As demonstrated by previous studies, specific knockout of the Piezo2 channel in Aδ and C fibers led to prolonged contraction intervals and marked attenuation of urethral reflexes in mice (Marshall et al., 2020). Aside from that, clinical studies have found that patients with loss-of-function mutations in Piezo2 exhibit lowered bladder filling sensation, along with symptoms such as urinary incontinence and nocturnal enuresis (Marshall et al., 2020). As suggested by these findings, the Piezo2 channel serves as a pivotal mechanoreceptor for sensory neurons to detect bladder filling under physiological conditions. For this reason, a plausible hypothesis is that the upregulation of Piezo2 expression reinforces the sensitivity of sensory neurons to intravesical mechanical stimuli, thereby overactivating the micturition center and inducing IC/BPS. In this case, we provide direct evidence to support this hypothesis. Consistent with previous reports (Liu et al., 2023), we found that Piezo2 expression in sensory neurons was functionally upregulated in CYP-induced IC/BPS model rat. Knockdown of Piezo2 via intrathecal injection of ASO considerably lessened c-Fos expression in the spinal dorsal horn, suggesting lowered activation of spinal neurons triggered by afferent signaling. Correspondingly, the lower urinary tract function of rats with IC/BPS was improved. We did not further explore the mechanisms underlying the upregulation of Piezo2 channel expression in sensory neurons. As demonstrated by a recent study, hyperactivation of the insulin-like growth factor-1 (IGF-1) signaling pathway mediates the upregulation of Piezo2 in peripheral sensory neurons during tactile hypersensitivity in a mouse model of autism spectrum disorder (Zhai et al., 2025). IGF-1 signaling is believed to reinforce the sensitivity of DRG neurons under inflammatory conditions (Zhang et al., 2014), while RNA sequencing has revealed that CYP induces the enrichment of inflammatory pathways in bladder sensory nerves (Chen B. et al., 2024). For this reason, the IGF-1/Piezo2 axis may be involved in the CYP-induced enhancement of bladder afferent activity. Our findings offer brand new insights into the pathological mechanisms of IC/BPS, and Piezo2-targeted strategies may represent a novel approach for the clinical treatment of IC/BPS.

Electroacupuncture is universally employed in clinical practice in China, including for the treatment of lower urinary tract disorders, yet its underlying fundamental principles and neural mechanisms remain unclear. In such case, we conducted a preliminary exploration to elucidate the mechanism by which EA treats IC/BPS. Our results demonstrated that EA downregulates Piezo2 expression in sensory neurons and decreases the number of spinal neurons activated by afferent signals, which may contribute to the modulation of bladder function via ascending micturition neural circuits. These findings are consistent with those of a recent study (Guo et al., 2022), suggesting that Piezo2 may contribute to a broader range of EA effects. Nonetheless, attributable to the deficiency of a Piezo2-specific agonist, it is challenging to confirm the specificity of Piezo2 in this regulatory pathway. We observed that the therapeutic efficacy of combined intervention with Piezo2 knockdown and EA was not significantly superior to that of monotherapies. One plausible explanation is that EA fails to elicit any additional therapeutic benefits owing to the loss of its downstream target. Nevertheless, this phenomenon may also stem from the therapeutic ceiling effect. Accordingly, Piezo2 knockdown alone cannot validate that Piezo2 is an indispensable mediator for EA to exert its therapeutic effects. It remains ambiguous how EA induces changes in bladder sensory neurons. In recent times, a mechanism has been proposed by which neuromodulation treats OAB by inhibiting bladder afferent signals, promoting spinal cord defense reflexes, and regulating non-specific supraspinal regulatory circuits (Krhut et al., 2025). The S1 sacral nerve, which contains both afferent and efferent components of the bladder, passes through the deep muscle tissue of the second posterior sacral foramen in rats. We put forth a hypothesis EA may exert a direct stimulatory effect on this nerve, a principle analogous to that of sacral nerve stimulation for the treatment of IC/BPS. On top of that, there is supplementary evidence that the 10 Hz EA frequency we selected has been proven to produce optimal effects in regulating bladder function in rats via sacral nerve stimulation (Su et al., 2012, 2013, 2016; Potts et al., 2019). Our study may also offer profound insights into elucidating the mechanism underlying the treatment of IC/BPS with sacral nerve stimulation. Recent studies have underscored the pivotal role of the somatosensory-vagus nerve reflex pathway in mediating the anti-inflammatory and gastric function-regulating effects of EA at Zusanli (Liu et al., 2021; Dong et al., 2025). This reflex relies on specific somatosensory neurons innervating the deep fascial tissues to transmit EA stimulation signals to the dorsal motor nucleus of the vagus nerve (Liu et al., 2021; Dong et al., 2025). In our study, the non-acupoint control group received acupuncture without penetrating the second posterior sacral foramen. This further suggests that the deep, rather than superficial, tissues of BL32 mediate the regulatory effects of EA on lower urinary tract function in rats with IC/BPS. It is particularly noteworthy that EA and non-acupoint EA groups differ not only in acupoint location and tissue penetration depth, but also in stimulation magnitude of deep sacral tissues and sacral nerve-related structures. Accordingly, the current experimental design cannot fully dissociate acupoint specificity from deep sacral foramen stimulation effects. Nevertheless, as evidence supporting whether the efferent components of the vagus nerve innervate the bladder remains lacking, other specific brain-somatic neural circuits may account for mediating EA effects. Apart from that, a preprint study has identified a population of polyorganic sensory neurons that can respond to both bladder and colonic signals (Kim et al., 2025). On that account, it is worthwhile to probe into whether a direct “somatosensory-visceral sensory” circuit exists to mediate EA effects.

## Data Availability

The raw data supporting the conclusions of this article will be made available by the authors, without undue reservation.

## Supplementary material

Supplementary Figure 1 **(a)** Comparison of body weight before and after injection of different doses of CYP. **(b,c)** Changes in the thickness of the bladder urothelium **(b)** and degree of bladder fibrosis in rats **(c)** induced by different doses of CYP, *n* = 6. **(d)** Representative images of H&E staining of rat bladders after administration of different doses of CYP. Red arrows indicate lamina propria edema, and the area outside the yellow dashed line represents the urothelium. **(e)** Representative images of Masson staining of rat bladders after administration of different doses of CYP. **(f,g)** Representative images of Piezo2 immunofluorescence staining in L6 lumbar DRG tissue sections of rats in control, CYP, CYP+ASO Piezo2, and CYP+Mismatch group **(f)**, with quantitative analysis **(g)**. **(h-l)** Representative images of void spot assay in rats from control, CYP, CYP+ASO Piezo2, and CYP+Mismatch group **(h)**, and comparative analyses of void frequency **(i)**, void volume **(j)**, average void volume **(k)**, and void frequency in the middle 50% of the cage **(l)**, *n* = 6. For immunofluorescence staining and RNAscope, three non-consecutive sections per rat were counted, *n* = 6. All data are expressed as mean ± SEM. Statistical significance in the graphs is indicated as **P* < 0.05, ***P* < 0.01, ****P* < 0.001, *****P* < 0.0001.

Supplementary Figure 2 **(a–e)** Representative images of void spot assay in rats from control, CYP, CYP+EA, and CYP+NEA group **(a)**, and comparative analyses of void frequency **(b)**, void volume **(c)**, average void volume **(d)**, and void frequency in the middle 50% of the cage **(e)**, *n* = 6. **(f,g)** Representative images of Piezo2 immunofluorescence staining in L6 DRG sections of rats in control, CYP, CYP+EA, and CYP+NEA group **(f)**, and quantitative analysis **(g)**. All data are expressed as mean ± SEM. Statistical significance in the graphs is indicated as **P* < 0.05, ***P* < 0.01, ****P* < 0.001, *****P* < 0.0001.

Supplementary Figure 3 **(a–g)** Example pressure traces of cystometry in rats from control, CYP, CYP+EA, CYP+ ASO Piezo2, and CYP+ASO Piezo2+EA group **(d)**, and comparative analyses of contraction intervals **(b)**, peak bladder pressure **(c)**, bladder pressure baseline **(d)**, pre-contraction pressure **(e)**, bladder compliance **(f)**, and bladder contractions **(g)**, *n* = 6. **(h–l)** Representative images of void spot assay in rats from each group **(h)**, and comparative analyses of void frequency **(i)**, void volume **(j)**, average void volume **(k)**, and void frequency in the middle 50% of the cage **(l)**, *n* = 6. All data are expressed as mean ± SEM. Statistical significance in the graphs is indicated as **P* < 0.05, ***P* < 0.01, ****P* < 0.001, *****P* < 0.0001.
